# Single neuron responses underlying face recognition in the human midfusiform face-selective cortex

**DOI:** 10.1038/s41467-023-41323-5

**Published:** 2023-09-13

**Authors:** Rodrigo Quian Quiroga, Marta Boscaglia, Jacques Jonas, Hernan G. Rey, Xiaoqian Yan, Louis Maillard, Sophie Colnat-Coulbois, Laurent Koessler, Bruno Rossion

**Affiliations:** 1 Hospital del Mar Research Institute (IMIM), Barcelona, Spain; 2 Institució Catalana de Recerca i Estudis Avançats (ICREA), Barcelona, Spain; 3 Centre for Systems Neuroscience, University of Leicester, Leicester, UK; 4 Ruijin hospital, Shanghai Jiao Tong university school of medicine, Shanghai, China; 5 Université de Lorraine, CNRS, CRAN, F-54000 Nancy, France; 6 Université de Lorraine, CHRU-Nancy, Service de Neurologie, F-54000 Nancy, France; 7 Université de Lorraine, CHRU-Nancy, Service de Neurochirurgie, F-54000 Nancy, France

**Keywords:** Extrastriate cortex, Sensory processing

## Abstract

Faces are critical for social interactions and their recognition constitutes one of the most important and challenging functions of the human brain. While neurons responding selectively to faces have been recorded for decades in the monkey brain, face-selective neural activations have been reported with neuroimaging primarily in the human midfusiform gyrus. Yet, the cellular mechanisms producing selective responses to faces in this hominoid neuroanatomical structure remain unknown. Here we report single neuron recordings performed in 5 human subjects (1 male, 4 females) implanted with intracerebral microelectrodes in the face-selective midfusiform gyrus, while they viewed pictures of familiar and unknown faces and places. We observed similar responses to faces and places at the single cell level, but a significantly higher number of neurons responding to faces, thus offering a mechanistic account for the face-selective activations observed in this region. Although individual neurons did not respond preferentially to familiar faces, a population level analysis could consistently determine whether or not the faces (but not the places) were familiar, only about 50 ms after the initial recognition of the stimuli as faces. These results provide insights into the neural mechanisms of face processing in the human brain.

## Abstract (web summary)

Face selectivity in the human brain has mainly been assessed with neuroimaging methods. Here, the authors perform single-neuron recordings in the human midfusiform gyrus, finding a higher number of neurons responding to faces than to places.

## Introduction

Converging evidence has shown that visual object recognition is processed along the ventral visual pathway^1–3^. In macaque monkeys, face-selective neurons have long been identified in the highest stages of this pathway, i.e., the infero-temporal cortex^4,5^. More recent studies, combining functional magnetic resonance imaging (fMRI) with neurophysiological recordings, have described a number of face-selective patches containing almost exclusively neurons that respond selectively to faces^6,7^. In humans, for nearly three decades, neuroimaging studies have consistently shown larger responses to faces than to other visual categories (i.e., face-selective responses) in the ventral occipito-temporal cortex (VOTC), with a peak of activation in the lateral portion of the midfusiform gyrus (the “Fusiform Face Area”, “FFA”)^8–11^. The key role of this region in face recognition is further supported by intracranial electrophysiology studies^12,13^, face-selective identity recognition impairments elicited by focal lesions^14^ and face-related perceptual effects following direct electrical stimulation^15,16^. There are basically two different mechanisms that can produce the observed fMRI responses: i) as in the monkey face patches^6,11^, the human midfusiform region may have a majority of neurons that respond selectively to faces (with other neurons being selective for other category of stimuli), and ii) an alternative hypothesis is that the human midfusiform face activations observed with fMRI are generated by broadly tuned neurons that respond strongly (but not exclusively) to faces and have also weaker responses to other category of stimuli.

In addition to the lack of a mechanistic understanding of the neuronal responses giving rise to face-selective neural responses in humans, the role of the midfusiform gyrus in the recognition of familiar faces is controversial, with neuroimaging studies reporting mixed results^17–19^. Moreover, there is very little work comparing neural responses to familiar versus unknown faces in monkeys^20,21^ and, considering the thousands of familiar individuals that humans know by their faces^22^, testing face familiarity in humans may reveal unique outcomes. According to a prevalent view, the human face-selective midfusiform region holds similar representations of familiar and unknown faces, which are distinguished at later stages of processing in the temporal lobe^18,23^. Alternatively, the representation of familiar and unfamiliar faces may already differ in this region, shortly after, or even concurrently to face-selectivity^17^. To address these issues, and using the unique opportunity to record from multiple individual neurons in humans, in the present study we investigated: first, the neuronal responses underlying the human midfusiform face-selective activations, and second, whether the midfusiform neuronal responses hold information about the long-term familiarity of the face identities.

## Results

**Fig. 1:**
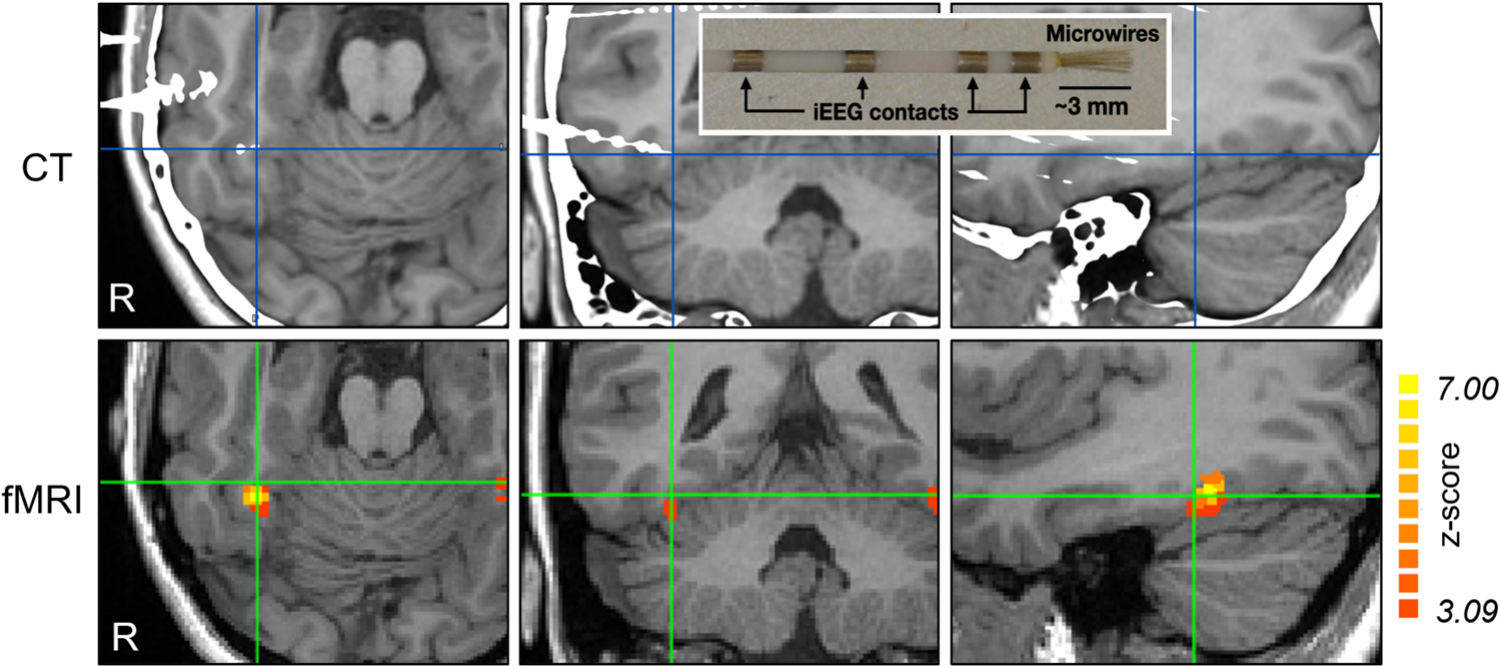
Electrode location in one patient. Axial, coronal, and sagittal views of a CT-MRI coregistration, showing the estimated position of the microwires (at ~3 mm from the tip of the intracerebral electrode) marked with a cross, and functional Magnetic Resonance Imaging (fMRI) showing the midfusiform gyrus activation. The colorbar on the right denotes the normalized (*z*-score) activation compared to baseline (see “Methods” section). The inset on the top right shows the depth electrodes with microwires used for single neuron recordings. Source data are provided as a Source Data file.

We recorded from 78 units (33 single-units and 45 multi-units) in the midfusiform gyrus of 5 patients implanted with intracerebral electrodes for refractory epilepsy pre-surgical exploration. During the experiment, the subjects viewed pictures corresponding to four categories: familiar faces, unknown faces, familiar places, and unknown places (Fig S1). Figure 1 shows the face-selective fMRI activations for one of the patients, together with the placement of the intracerebral electrode, ending in this region. For all patients, we verified that the electrodes for single neuron recordings ended in the midfusiform gyrus, although they had variable locations with respect to the peak of the face activations (Fig. S3). For this reason, we refer to responses in the face-selective midfusiform gyrus and not in the FFA (or even more specific functional subregions such as pFus-faces/FFA1, mFus-faces/FFA2;^24^). Yet, importantly, for all five patients, the regions in which we performed the recordings were face-selective, as determined from two independent fMRI and Local Field Potential (LFP) face localizer experiments (Fig. S4). Moreover, we found units responding to faces and places in all patients (Fig. S5), and the proportion of both face and place responses was statistically the same across patients (*χ*^2^ test, *p* = 0.2 and *p* = 0.4, respectively).

### Midfusiform neurons have a variety of responses

**Fig. 2:**
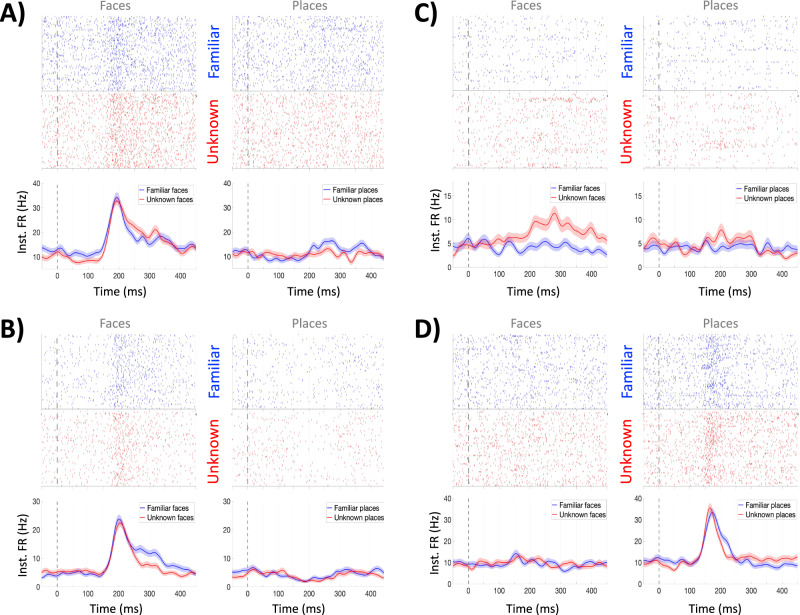
Example of 4 neuronal responses in the face-selective midfusiform cortex. **A** A single neuron responding to pictures of faces (and not to pictures of places), without a significant difference between the responses to familiar and unknown faces (two-sided *t*-test). **B** A single neuron also showing face-responses but with a significantly larger response to the familiar faces (*p* < 0.05, two-sided *t*-test). For this neuron, there was also a less pronounced but significant decrease in firing in response to pictures of places (*p* < 0.05, two-sided *t*-test). **C** A face-responsive single neuron, with a significantly larger response to unknown faces (*p* < 0.05, two-sided *t*-test). This modulation, however, is very different to that of the previous neurons, showing a more variable response pattern, responding selectively to a few unknown faces. **D** A multi-unit with a significant response to places, without modulation by familiarity. The shaded areas represent SEM. Source data are provided as a Source Data file.

All patients showed units responding to faces and places. Four exemplary units are displayed in Fig. 2, showing a variety of responses to faces, to places and to both category of stimuli. Altogether, from the 78 detected units, 43 (55%) were responsive (paired t-test, *p* < 0.05) and, of these: 19 responded only to faces (as in Fig. 2a, c), 7 responded only to places (as in Fig. 2d), and 17 responded to both faces and places (as in Fig. 2b)—i.e. 36 (46%) of the recorded units showed a response to faces and 24 (30%) to places (see Table S1). The number of units responding to faces and to places was significantly larger than chance (*p* < 10^−25^ and *p* < 10^−12^, respectively, binomial test), and there were more units responding to faces than to places (*p* < 10^−3^, binomial test). Of the 36 responses to faces, 25 were enhanced responses and 11 were suppressed, whereas of the 24 responses to places, 13 were enhanced, and 11 where suppressed. Eleven (30%; 9 enhanced and 2 suppressed) of the face-responsive units showed a significant familiarity modulation (*t*-test, *p* < 0.05), whereas a familiarity modulation was found for 6 (25%; 5 enhanced and 1 suppressed) place-responsive units (*t*-test, *p* < 0.05). Altogether, we could not identify any obvious visual feature in our stimulus set (Fig. S1B) that triggered the unit responses. As shown with the examples of Fig. 2, and as also shown in the next section with a wide variety of responses, in terms of their strength, latency, selectivity, and familiarity, different neurons had different tuning preferences given by non-trivial features of the stimuli.

### Similar average responses to faces and places

**Fig. 3:**
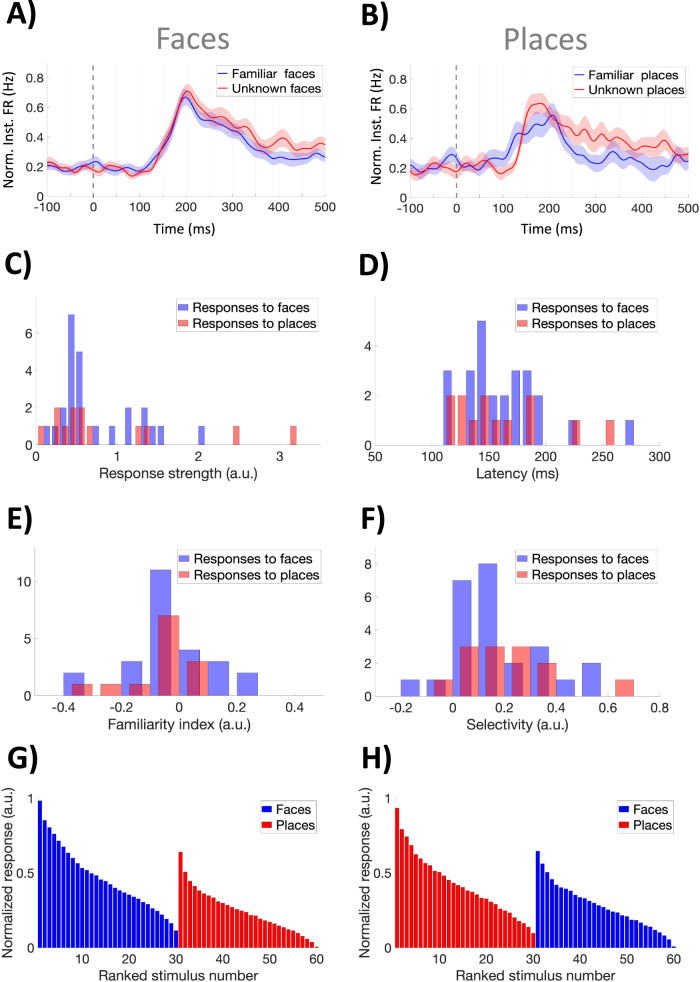
Characterization of single neuron responses. **A**, **B** Normalized grand average of enhanced responses to faces (*N* = 25) and places (*N* = 13). In both cases, the responses to the familiar and unknown stimuli were very similar. **C**, **D** Distribution of response strength and latency, with no significant differences between the responses to faces and places (**C**: bin size=0.1; **D**: bin size=10). **E** Familiarity index values for the place and the face responses. Values are centered around zero—i.e. not showing any clear tendency to respond to familiar or unknown pictures—and, as before, there were no differences between the face and place responses (**E**: bin size=0.1). **F** Selectivity values, also showing no differences between the face and place responses- (**F**: bin size=0.1). **G**, **H** Average of the normalized ranked responses for the face- and place-responsive units, respectively. In both cases, a graded pattern of responses was observed, with face-responsive neurons also responding to places, and vice versa. The shaded areas represent SEM. Source data are provided as a Source Data file.

Figure 3a, b show the normalized grand averages for the enhanced responses to faces (*N* = 25) and places (*N* = 13). (Analogous results were obtained for the suppressed responses and are shown in Fig. S6). We should clarify that we did not find any systematic trend in the responses across trials, showing adaptation or facilitation effects, for any of the category of stimuli (familiar and unknown faces and places; see Fig. S7). Therefore, when referring to familiarity effects, we refer to the responses to the pictures initially classified as familiar (or unknown; see “Methods” section) and not to how pictures may have become more familiar after several presentations.

For both places and faces, in the grand averages we observe similar responses to the familiar and the unknown pictures. To quantify this observation, we calculated the strength and latency of the individual responses (see “Methods” section). For each responsive neuron, the strength was normalized to the baseline firing activity (see “Methods” section and Fig. S11). There were no significant differences in the strength of the face and place response distributions (Fig. 3c; Wilcoxon rank-sum test); in addition, for both faces and places there were no significant differences between the familiar and unknown stimuli (Wilcoxon rank-sum test). Figure 3d shows the response onset latencies, which varied mostly between 100 and 200 ms, and were not significantly different between the face and place responses (median for face responses: 156 ms; median for place responses: 150 ms; Wilcoxon rank-sum test), or between the familiar and unknown stimuli in each case (Wilcoxon rank-sum test). Altogether, given that the responses to faces and places were not significantly different, the larger responses to faces observed at the population level in the midfusiform gyrus can be attributed to a larger proportion of neurons responding to faces in this region.

In the above analyses, we do not observe major differences between the responses to familiar and unknown faces or places. To further quantify whether midfusiform units tend to respond preferentially to familiar stimuli, we defined a familiarity index (*FI*), which gives positive (negative) values for units responding more strongly to familiar (unknown) stimuli, and values around zero for those responding similarly to both types of stimuli (see “Methods” section). Figure 3e shows the distributions of the familiarity index values, which were not significantly different for the face and place responses (Wilcoxon rank-sum test) and had values centered around zero (for each distribution the median was not significantly different from zero; sign test), meaning that, overall, the familiar and unknown stimuli elicited similar activations.

As in previous work^25^, to study the selectivity of the responses we used a measure that is independent of any threshold for defining responsiveness, and gives values around 0 for non-selective neurons and close to 1 for the selective ones (see “Methods” section). In Fig. 3f we observe that the responses of midfusiform units had a wide range of selectivity values, which were not significantly different between the face and the place responses, or between the familiar and unknown stimuli in each case (Wilcoxon rank-sum test). Considering our responsiveness criterion (see Methods), the responsive neurons responded to 15% of the presented stimuli on average. To visualize the overall selectivity of these neurons, in Fig. 3g, h we show the ranked normalized responses for the face and place selective units, respectively. In these plots we observe a gradual decrease of the responses, showing a graded tuning, as has been described in object and face-selective regions of the monkey visual cortex^1,5,7^ and in contrast to the nearly binary coding by downstream neurons in the human hippocampal formation^26^. Moreover, although with a lower strength, the face-responsive units also responded to some of the places (and vice versa), with the maximum response to the non-preferred stimulus category reaching about 60-70% of the maximum value for the preferred stimulus category.

### Coding of face familiarity

**Fig. 4:**
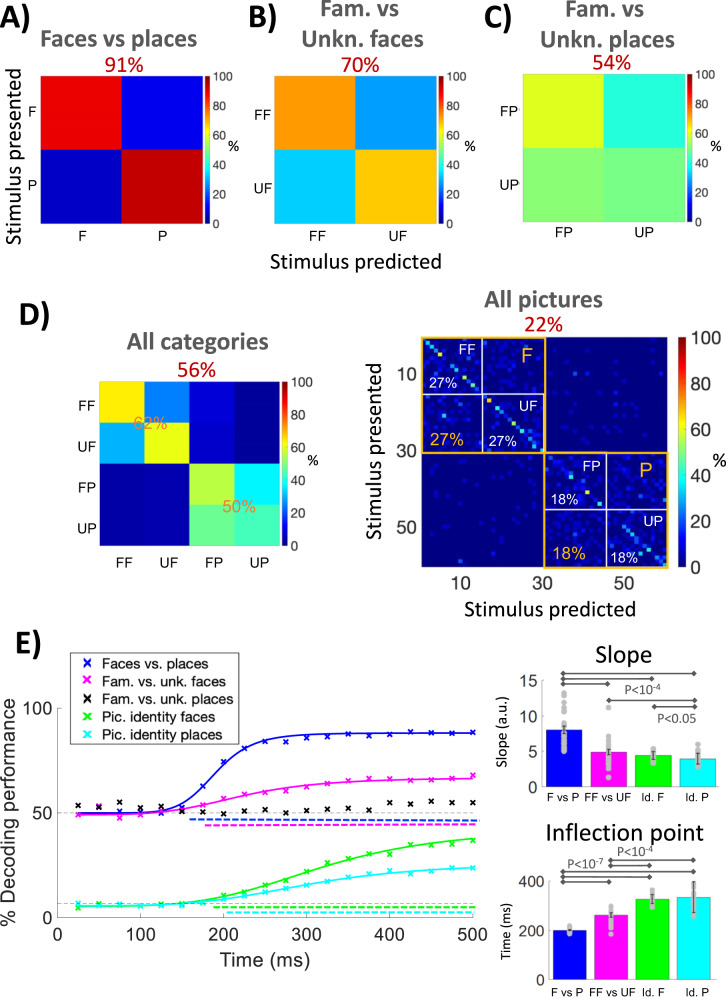
Population decoding results. **A** Decoding of whether the picture was a face (F) or a place (P), using all responsive units (*N* = 43); (**B**) decoding of whether the picture was a familiar face (FF) or unknown face (UF), using face-responsive units (*N* = 36); (**C**) decoding of whether the picture was a familiar place (FP) or unknown place (UP), using place-responsive units (*N* = 24). In all cases, the overall decoding performance is shown on top. **D** decoding of all 4 categories (familiar face, unknown face, familiar place, and unknown place) using all responsive units (left), and decoding of the pictures presented using all responsive units (right). Decoding performance was similar for familiar and unknown stimuli (for both faces and places) and was higher for faces (27%) compared to places (18%). **E** Cumulative decoder using all responsive units and discriminating: faces vs. places, familiar vs. unknown faces, familiar vs. unknown places (not significant), and the picture identity of the face and place stimuli. The solid lines mark the corresponding logistic regressions (see “Methods” section). The top and bottom dashed horizontal lines mark the chance level for the binary decisions (face vs. place; familiar vs. unknown face; familiar vs. unknown place) and discrimination of face/place identify, respectively. The colored dashed lines below the chance levels show the time periods where each decoder is significantly larger than chance (permutation test, *p* < 0.05). We observe different time profiles of the decoders, with the decoding of faces vs. places having an earlier inflection point and higher slope compared to the other ones (right panels; two-sided *t*-test; *N* = 24). The inflection point was also earlier for the familiar vs. unknown face decoder compared to the face and place picture identification. Source data are provided as a Source Data file.

Given the mixed results obtained with the analysis of individual unit responses, we carried out a population decoding analysis—predicting in each trial the stimulus being displayed based on the neurons’ responses—to assess whether the midfusiform neurons provide a reliable discrimination between faces and places, on the one hand, and of familiar versus unknown faces, on the other. Considering all 43 responsive units (similar results were obtained when using all the recorded units), the mean decoding performance discriminating the presentation of faces *vs*. places was much larger than chance (91%, *p* < 10^−87^; permutation test; Fig. 4a). Likewise, taking only the 36 face-responsive units, the discrimination between familiar and unknown faces was also significantly larger than chance (70%, *p* < 10^−11^; permutation test; Fig. 4b), but the discrimination between familiar and unknown places, considering the 24 place-responsive neurons, was only marginally significant (54%, *p* = 0.085; permutation test; Fig. 4c). Considering all responsive units and all picture categories (familiar or unknown faces or places), the decoder was able to distinguish whether the pictures corresponded to a face or a place (Fig. 4d). In fact, in only 9% of the cases a face was confused with a place—a much lower rate than that expected by chance (*p* < 10^−92^; permutation test). As before, the decoder could predict whether the stimulus corresponded to a familiar or unknown face, but it confused familiar vs. unknown places.

Figure 4d shows the performance obtained when decoding the 60 pictures presented using all responsive units. We observe a clear pattern along the diagonal, with an average hit rate of 22%, significantly higher than chance (1/60 ~ 1%; *p* ~ 0; permutation test). In particular, the average number of correct predictions for faces (27%) was significantly higher than for places (18%; *p* < 0.05, *t*-test) and, for both faces and places, there were no significant differences in the predictions of the familiar versus the unknown individual pictures (*t*-test). The high decoding performance obtained for the individual pictures raises the concern that the familiarity information obtained for faces (Fig. 4b) could be a byproduct of the relatively high discriminability of the individual faces—i.e. midfusiform neurons could just discriminate individual faces without providing any specific information about their familiarity. To address this issue, we repeated the decoding analysis of familiar versus unknown faces but shuffling the familiar/unknown labels of each picture, and found that the performance obtained was significantly lower than when not shuffling the picture labels (*p* < 0.005, permutation test), thus confirming that these units contain information about both the familiarity of the faces and picture identity. Likewise, when shuffling the ‘face’/‘place’ labels of the stimuli, we obtained a significantly lower performance decoding faces versus places, compared to the one obtained without shuffling the labels shown in Fig. 4a (*p* < 10^−32^). However, reshuffling the familiar/unknown labels of the place pictures did not significantly change the decoding performance (*p* = 0.6) and therefore, the marginally significant performance when decoding familiar vs. unknown places was likely due to the discriminability of one or relatively few individual places. To further confirm these results, we also performed a K-fold decoding crossvalidation – excluding from the training set all the trials corresponding to the picture being tested – and obtained qualitatively similar results (Fig. S8).

### Three face-related discriminations at the population level

From the decoding results described above, we infer that there are 3 discriminations taking place at the population level in the face-selective midfusiform gyrus: face detection, familiarity face recognition, and picture identification. To investigate the time profile of these discriminations, we carried out a decoding analysis in which we successively expanded the time window considered (i.e. the information that would in principle be accessible to downstream neurons integrating inputs from the midfusiform population), predicting whether the picture was a face or a place, a familiar or an unknown face, and the identity of the face (place) picture. Figure 4e shows that the decoding curves had different time profiles, with the face vs. place discrimination showing an earlier increase compared to the other curves, and an inflection point occurring significantly earlier and with a significantly higher slope (left panels of Fig. 4e). The inflection point was also significantly earlier in the familiar vs. unknown face curve compared to the face and the place picture identity decoding curves. Both a time-resolved decoding analysis using a sliding window, and a cross-time decoding analysis considering different time windows in the training and test sets, gave qualitatively similar results (shown in Figs. S9 and S10, respectively).

## Discussion

Contrasting with the vast neuroimaging and electrophysiological evidence characterizing different aspects of face-selective activity in the human midfusiform gyrus^8–13,18,19^, the neural mechanisms producing such activations have remained elusive, due to the lack of single neuron recordings in this region (for a few recent studies recording face cells in the human VOTC, see ref. ^27^ and the single cases reported in refs. ^28,29^). Neurons responding to faces have long been identified in the temporal cortex of macaque monkeys^1,4,5^, with fMRI-defined face-selective clusters containing nearly exclusively neurons responding to faces^6,7^ (but see ref. ^30^) and suggesting that a similar type of neuronal responses may underlie the fMRI activations observed in humans^11^. Here, recording directly from individual neurons in the face-selective human midfusiform gyrus, we found that: First, in humans there is also a tendency to find face-responsive neurons. Second, midfusiform neurons did not fire only to faces, as 30% of the recorded units fired to places. Third, the responses to faces and places in face- and place-responsive units, respectively, were statistically the same in terms of their strength, latency and selectivity. These results suggest that the stronger responses to faces compared to non-face objects found with neuroimaging, scalp and intracranial EEG recordings in humans^9,10,12^ are due to a higher proportion of face-responsive neurons in this region rather than broadly tuned neurons responding stronger to faces compared to other category of stimuli.

Although in our study there was a tendency to find face-responsive units in the fusiform gyrus (46%), the proportion of these units was not as high as reported in macaques (~90% or more)^6,7^. In this respect, our results are actually closer to those in non-human primate studies that sampled more broadly within face-selective clusters (ref. ^30^, see also ref. ^31^ for intermediary values). The lack of nearly exclusive face-selective responses reported here could be due to differences across species and regions (i.e., recordings in monkeys were performed in the lateral temporal cortex, whereas here we recorded ventrally in the fusiform gyrus – a region that is not present in macaques and is specific to hominoids/apes^32^). Another possibility is that this structure is indeed specialized for faces but also represents other non-face stimuli, given the richness of perceptual discriminations across a vast number of meaningful stimuli categories determined essentially by natural experience (and not potentially biased by extensive reward-driven training with particular sets of stimuli). However, it should also be noted that, due to clinical constrains, the placement of the electrodes varied from patient to patient (see Fig. S3) and was not necessarily at the peak of the face-selective activations (as defined from the independent fMRI and LFP face-localizer experiments), which is the area typically targeted in monkeys^6,7^. This might be particularly relevant, considering the changes in the tuning of single neurons in a specific area reported at the submillimeter scale^33^. However, it is important to mention that, in spite of the variable localization of the electrodes within the midfusiform gyrus, a similar proportion of face and place responses was found for all patients in this study (Fig. S5). Further studies including more subjects with recording locations close to or at the peak of the face-selective activations will be required to determine if the finding of nearly-exclusive face-selective neurons, as in the monkeys’ face patches, is also present in the human brain.

Due to lack of direct single neuron recordings, the role of the midfusiform region in coding long-term face familiarity has been controversial^11,18,19,34^, with some neuroimaging studies finding different activations for familiar compared to unfamiliar faces^8,17,35–38^ but the majority reporting no familiarity effects in the fusiform gyrus^34,39–45^ (among others). Humans are familiar with thousands of faces^22^, and familiar faces have a special status, being associated with rich semantic networks^22,23,46^. Our results show that neurons in the midfusiform region have an equal tendency to respond to familiar and unknown faces, and no significant differences were observed in the grand averages, or in the average strength, latency, and selectivity of the responses. However, given that about 30% of the face responsive units showed a familiarity modulation (with a larger response either to the familiar or the novel faces but, as mentioned above, without showing a difference in the averages) when performing a single-trial decoding analysis of the midfusiform population activity — exploiting the single neuron resolution of our recordings and extracting information beyond the one provided by grand averages or non-invasive recordings — the decoder gave reliable information about whether the pictures corresponded to a familiar or unknown face. This result indicates that neurons in this relatively posterior region within the cortical face network respond beyond mere physical features of the faces and are already shaped by the subjects’ previous experience, i.e., making them familiar with certain faces identities —an information that could be read by downstream anterior temporal lobe areas to differentially process familiar faces and their meaning^23,47^.

Overall, the single-trial population analysis led to identifying three face-related discriminations taking place in the midfusiform gyrus: the categorization of the stimulus as a face, familiarity recognition, and (face and place) picture identification. While the discrimination of the stimulus as a face saturated early, followed by the discrimination of its familiarity, the discrimination of the individual (face and place) pictures kept increasing in the time window considered, reaching a performance more than 10 times larger than what would be expected by chance (Fig. 4d). This contrasts to relatively small or no increases of performance above chance reported with fMRI multivoxel pattern analyses in face-selective cortex^11,48,49^, which led some authors to argue that the midfusiform cortex may merely detect faces, with the identity of the faces being recognized in more anterior cortical regions^48^. Instead, our results at the level of individual neurons agree with the view derived from fMRI and iEEG adaptation paradigms, showing that the face-selective midfusiform cortex is involved in both face detection and face individuation^50,51^, although it should be noted that we did not test different views of the same faces to evaluate generalization in individual face discrimination. The finding of three different time profiles of face-related activations (face detection, followed by familiarity recognition, and then followed by face image identification) is in line with a coarse-to-fine processing of information, with e.g., an earlier representation of the age and gender of the faces before identity information^52^.

The human medial temporal lobe (MTL), and within it, the hippocampus, receives direct projections from high-level visual areas^53^ and has neurons with very selective and invariant responses to specific persons or places^54,55^. Compared to the upstream midfusiform neuron responses reported here, hippocampal neuron responses occur much later (i.e. with an onset of ~300 ms compared to ~150 ms in the midfusiform). This latency gap suggests the existence of much further neocortical processing – possibly to derive stimulus meaning^54,56^ – before the neural signals reach the hippocampal memory system. Moreover, human hippocampal neurons have a lower baseline activity (2.2 Hz^57^ vs. 4.8 in the fusiform gyrus; see Fig. S11), nearly binary tuning curves (rather than graded responses, as shown here in the midfusiform), and respond mainly to familiar stimuli^54^. Another notable difference is the much lower selectivity we found in the fusiform gyrus, with responsive neurons being on average activated by 15% of the stimuli, compared to the selectivity of hippocampal neurons, responding on average to 1.7% of the stimuli^57,58^. Future research should provide more insights into how the representation of faces, involved in face recognition in the midfusiform gyrus, leads to a selective and invariant representation of familiar persons, for memory functions in the MTL^54^.

## Methods

Subjects performed a main experiment in which they were shown pictures of familiar and unknown faces and places, while we recorded the activity of multiple single neurons in the midfusiform gyrus. Moreover, in order to determine the location of the electrodes with respect to the face-selective midfusiform region, we performed two additional, independent experiments with the same frequency-tagging approach to isolate face-selective voxels in fMRI^59^ (see section “FMRI face localizer” below) and face-selective local field potentials^13^ (see section “LFP face localizer” below). Except explicitly mentioned otherwise, all quantifications described in this study (e.g., responsiveness, selectivity, etc.) correspond to the unit responses of the main experiment, as described in the following sections.

### Main experimental design

The data comes from 7 sessions in 5 patients with pharmacologically intractable epilepsy (all right-handed, 4 females, 24−42 years old), who were implanted with depth electrodes for about a week to determine the seizure onset zone for possible subsequent surgical resection^54^, at the epilepsy unit of the University Hospital of Nancy, France. All patients gave written informed consent and the study, including number of patients, selection criteria, and electrode implantation locations (REUNIE, trial No. 2015-A01951-48, ClinicalTrials.gov identifier NCT02877576) was approved by the ethical committee CPP Est III, No 16.02.01. The electrode locations were based exclusively on clinical criteria and were verified by CT fused with preoperative MRI. Here we report data from sites in the right (2 patients) and left (3 patients) face-selective midfusiform region. Each electrode probe had a total of 9 micro-wires at its end, 8 active recording channels and one reference (AdTech), protruding ~3 mm from the tip of the depth electrodes^54^. The differential signal from the micro-wires was amplified using a Blackrock system (NeuroPort Central Suite version 6.5.4), filtered between 0.3 and 7500 Hz and sampled at 30 kHz.

Subjects sat in bed, facing a laptop computer on which 60 images were presented for 500 ms, in pseudorandom order and without blanks in between, with a software written in Matlab (R2018a) that used the Psychophysics Toolbox. This sequence of picture presentations was triggered with a keypress by the patient, it lasted 30 s and was repeated 15 times. To ensure that they paid attention to the pictures, the patients had to indicate with a key press whether one of two color bars (either red or green), on the top and bottom borders of the pictures, changed color (Fig. S1a). The experiment lasted about 10 min in total.

The laptop was placed in a hospital tray, ~50–70 cm away from the patient, with each picture sustaining about 2 degrees visual angle from their view. In order to assess whether neurons respond preferentially to faces and how they encode familiarity, the pictures corresponded to 4 categories: 15 known faces (famous actors and politicians, in principle known to the patients), 15 unknown faces (local actors and politicians from Argentina, in principle unknown to the patients), 15 known places (Eiffel Tower, Arc de Triomphe, etc.), and 15 unknown places (local and mostly unknown scenes from UK towns; Fig. S1b). We contrasted faces to places because: (1) places are commonly contrasted to faces in human neuroimaging and electrophysiological studies^10^, and (2) familiar and unknown items can be easily selected for both types of stimuli to evaluate specific effects of face familiarity. To ensure that the patients were familiar with the people and places of the ‘known’ category compared to the ‘unknown’ one, they were asked to rank how much they knew the person/place of the pictures using a scale from 1 (completely unknown) to 7 (very familiar). Four of the five patients filled this questionnaire and for each of these patients we found a significantly higher score both for the faces and places of the ‘known’ category (in all cases *p* < 0.05; T-test). Rather than transforming the images in order to match their low-level visual features, which is prone to make the images look less natural compared to the original versions^60^, we chose a variety of images with overlapping distributions of low-level features in the 4 categories, with which we verified that the FFA neuron responses cannot be trivially attributed to low-level visual features (see Fig. S2).

### Statistics analysis

The significance of the single neuron responses was established using paired t-tests, comparing baseline with response time windows (see below), and for the main statistical analysis, the differences of the responses to the four categories were assessed using non-parametric Wilcoxon rank-sum tests (see below). No data were excluded from the analyses and no statistical method was used to predetermine sample size. The experiments were not randomized and the investigators were not blinded to allocation during experiments and outcome assessment.

### Spike sorting and responsiveness criteria

From the continuous wide-band data, spike detection and sorting was carried out using ‘Wave_Clus’, an unsupervised clustering algorithm^61,62^. Neurons were classified into single- or multi-units based on: (1) the spike shape and its variance; (2) the ratio between the spike peak value and the noise level; (3) the ISI distribution of each cluster; and 4) the presence of a refractory period for single-units; i.e., <1% spikes within <3 ms ISI^63^. Altogether we recorded a total of 78 units (33 single-units and 45 multi-units). The mean number of detected units per micro-wire was 1.39 (s.d.: 0.82).

For each stimulus we defined a “response window” between 100 and 400 ms from stimulus onset, and a corresponding “baseline window” between −100 and 100 ms. A unit was considered to be responsive if, either for the face or for the place stimuli, the average number of spikes in the response window was significantly different than the one in the baseline window, according to a paired t-test with *p* < 0.05. Considering the probability of observing a response to the face/place stimuli with a chance level of 0.05, we estimated the probability of obtaining N_F_ and N_P_ responses by chance (with *N*_F_ and *N*_P_ the number of face and place responses from the 78 recorded units, respectively) using a binomial test. For the face-responsive units, we assessed whether they showed a significant familiarity modulation, by comparing the responses to unknown versus known faces, and likewise, we compared the responses to unknown versus known places for the units responding to places (t-test, *p* < 0.05).

### Familiarity index

In order to quantify a tendency of the neurons to respond more to familiar or unknown pictures (faces or places), we defined a familiarity index as:

1 $${FI}=\frac{\left\langle {Response}\,{Familiar}\right\rangle -\left\langle {Response}\,{Unknown}\right\rangle }{\left\langle {Response}\,{Familiar}\right\rangle+\left\langle {Response}\,{Unknown}\right\rangle }$$

where $\left\langle {Response\; Familiar}\right\rangle$ and $\left\langle {Response\; Unknown}\right\rangle$ correspond to the mean response to the familiar and unknown stimuli (faces or places). Units with no familiarity modulation will have values around zero, whereas units with a larger response to familiar (unknown) pictures will tend to have positive (negative) values. The familiarity index values obtained for the face and the place responses were compared using a non-parametric Wilcoxon rank-sum test, and we further used a sign test to evaluate if the median of each of the distributions was significantly different from zero (thus showing a preference for familiar or novel pictures).

### Instantaneous firing rate and latency estimation

For each significant response, the response latency was estimated from the instantaneous firing rate, which was obtained by convolving the spike train with a Gaussian of 10 ms s.d. and then averaging across trials. The latency was then computed as the time where the instantaneous firing crossed the baseline plus 2 s.d. value (or minus 2 s.d. for suppressed responses) for at least 50 ms. For some responsive units, a response latency could not be calculated according to this criterion (i.e., the instantaneous firing rate did not cross the baseline plus 2 s.d. for more than 50 ms) and therefore these cases were not included in the latency distribution. Latency distributions were compared using a non-parametric Wilcoxon rank-sum test. For the grand average curves (Fig. 3a, b and S6a, b), each face/place response was normalized to have a maximum range (either for familiar or unknown faces/places) between 0 and 1 for enhanced responses, and 0 and −1 for the suppressed responses.

### Strength and selectivity

For each face- (place-) responsive unit, we calculated the normalized response strength as: ${RS}=\left\langle \frac{R-B}{B}\right\rangle$, where *R* are the responses to faces (places), *B* the baseline activity and $\left\langle \ldots \right\rangle$ denotes the mean across face (place) stimuli. We also estimated the selectivity of each responsive unit using a measure that it is not dependent on a responsiveness threshold, as used in previous works^25,64^. For this, we calculated the relative number of stimuli *R(T)* eliciting a response larger than a threshold *T* as:

2 $$R\left(T\right)=\frac{1}{N}\mathop{\sum }\limits_{i=1}^{N}\theta ({f}_{i}-T)$$

where *f*_*i*_ denotes the mean response to each face/place stimuli, *N (* = *30)* denotes the number of face/place stimuli, and $\theta \left(x\right)=1\, {for\; x} \, > \, 0$ and $\theta \left(x\right)=0\, {for\; x}\le 0$. Then, we defined the selectivity index as:

3 $$S=1-\frac{2}{M}\mathop{\sum }\limits_{j=1}^{M}R({T}_{j})$$

where ${T}_{j}={f}_{\min }+j({f}_{\max }-{f}_{\min })/M$ are equidistant thresholds between the minimum and maximum response, respectively, and *M* denotes the number of thresholds used (*M* = *1000*). This measure is independent of a particular threshold used to define responses, is close to 0 for uniformly distributed random responses, and approaches 1 the more selective the unit is. For more details and comparisons with other selectivity measures, see^25^. Statistical comparisons for both the response strength and the selectivity distributions were done using non-parametric Wilcoxon rank-sum tests.

### Decoding analysis

A linear Bayesian classifier was used to decode the pictures presented to the subjects based on the firing of the responsive units in the response window (from 100 ms to 400 ms after stimulus onset)^65^. One at a time, the spike count of each responsive unit in each trial was used to test the classifier, which was trained with the remaining trials (leave-one-out cross-validation). The decoding performance was estimated as the percentage of trials correctly predicted, and its statistical significance was assessed in comparison to the performances obtained with a population of 1000 surrogates created by randomly shuffling the trial labels.

The cumulative decoding performance was calculated from time zero until 500 ms after stimulus onset, in steps of 25 ms. Cumulative decoding performances, showing a sigmoidal increase of information until reaching saturation, were fitted with a 4-parameter logistic regression:

4 $$f\left(x\right)=d+\frac{a-d}{1+{\left(\frac{x}{c}\right)}^{b}}$$

where *a* and *d* are the upper and lower asymptotes, determined by the maximum and chance level decoding performance, respectively, *c* is the inflection point, and *b* the slope of the curve at the inflection point. The four coefficients were estimated with an iterative least squares estimation using the MATLAB function *nlinfit*. To compare the different cumulative decoding curves, we created 30 realizations with a random selection of half the number of units each, discarding 6 realizations in which the root mean square error of the fit of at least one of the cumulative curves was >0.1. The slope and inflection points of the curves were compared using an ANOVA test and *t*-test post-hoc comparisons. The statistical significance of decoding performance was established using a permutation test (comparing with the values of the 1000 surrogates shuffling labels, using a rank test).

### FMRI face localizer

To verify the position of the electrodes with respect to the face-selective midfusiform gyrus, patients performed an additional fMRI face-localizer experiment (independently from the experiments described above) within 4 weeks after the intracerebral electrode implantation. We used a frequency-tagging fMRI design, which provides high signal-to-noise ratio (SNR) and reliability to define brain regions selective to human faces versus non-face objects, independently of low-level visual cues^59^. In this paradigm, inspired from EEG frequency-tagging studies, images are presented at a fast 6 Hz rate, using different images of nonface objects (*N* = 188) displayed for 7 sec, interleaved with mini-blocks of 7 (out of 28) face images shown every 9 sec (i.e., 1/9 s = 0.11 Hz). Within a mini-block, the 7 face images are interleaved with six object images to avoid adaptation effects and increase SNR^59^. Since all face images are presented at the same 6 Hz base frequency rate as the nonface object images, neural activity that is selective to faces is expressed at 0.11 Hz in the frequency spectrum of the fMRI signal^59^. Images subtended a viewing angle of 8° and were back-projected onto a projection screen by an MRI-compatible LCD projector. Each fMRI sequence (run) lasted 333 sec. One to four sequences (runs, mean 2.5 ± 1 runs) were tested for each patient.

#### MR image acquisition

All patients were scanned at the University Hospital of Nancy, using a 3 T Siemens Magnetom Prisma system with a 64-channel head-neck coil. Anatomical images were collected using a high-resolution T1-weighted magnetization-prepared gradient-echo image (MP-RAGE) sequence (192 sagittal slices, voxel size = 1 mm isotropic; flip angle (FA) = 9 °, field of view (FOV) = 256 × 256 mm^2^, matrix size = 256 × 256). The acquisition time for the anatomical scan was about five min. Functional images were collected with a T2*-weighted simultaneous multi-slice echo planar imaging (SMS EPI) sequence (TR = 1500 ms, TE = 34 ms, FA = 72°, FOV = 240 × 240 mm^2^, voxel size = 2.5 mm isotropic, matrix size = 96 × 96, interleaved), which acquired 44 oblique-axial slices covering the entire temporal and occipital lobes.

#### fMRI Analysis

The functional runs were motion-corrected with reference to the average image of the first functional run using a 6-degree rigid body translation and rotation using a motion correction software (MCFLIRT; www.fmrib.ox.ac.uk/fsl), and spatially smoothed with a Gaussian kernel of 3 mm (FWHM; i.e. full width at half maximum). Linear trends from the preprocessed time series of each voxel were removed. The SNR of the face-selective response at 0.11 Hz was calculated for each voxel independently, as in previous studies^59^. The activation and deactivation of the neural responses at the stimulation frequency was defined by the phase of the BOLD responses^59^. To define face-selective activations, we averaged each voxel’s time series across all face runs (Fig. S3). A response was considered as face-selective if the *Z*-score at the frequency bin of face stimulation exceeded 3.09 (i.e., *p* < 0.001 one-tailed: signal>noise).

#### Visualization

The functional activation map across runs for each patient was registered with their high-resolution T1-weighted image. To assess the spatial relationship between face-selective activations at the microwire locations, the T1-weighted images were fused with the post-operative CT-scan, and the tip of the macroelectrodes, as they appear on the CT-scan, was manually detected, and we estimated the coordinate of the microwires to be at about 3 mm from the macro-electrode tip (marked with a cross in Fig. 1 and Fig. S3). Finally, we rendered the estimated microwire coordinates into the T1-weighted MRI co-registered with the functional activation maps.

### LFP face localizer

To further validate the location of the electrodes with respect to the face activation areas, a second independent experiment was performed, in which the Local field potential (LFP) activity was recorded from the microwires with a frequency-tagging face localizer that has been used and validated in previous studies^13^. Participants viewed continuous sequences of natural grayscale images of objects (*N* = 200) presented at 6 Hz, with images of faces (*N* = 50) shown every 5th stimulus (i.e., 1.2 Hz). A sequence lasted 70 s. Four participants viewed 8 sequences, and one viewed 6 sequences. Half of the sequences were typically run before the main experiment. In this paradigm (analogous to the frequency tagging paradigms described in the previous sections), common neural activity to faces and nonface objects is expressed at the 6 Hz base frequency rate (and harmonics, i.e., 12 Hz, 18 Hz, etc.), while neural activity that is selective to faces is expressed at 1.2 Hz and harmonics in the LFP frequency spectrum^13,66^.

#### LFP analysis

A fourth-order zero-phase low-pass filter at 300 Hz was applied to each sequence. Data segments were cropped to contain an integer number of 1.2 Hz cycles beginning 2 s after the onset of the sequence, until approximately 68 s, averaged in the time-domain separately for each participant and each active recording channel (8 channels) and Fast Fourier Transformed (FFT). The FFT spectrum was cut into 4 segments centered at the fundamental face frequency and 3 harmonics (1.2 Hz to 4.8 Hz). Amplitude values in these 4 segments were summed and averaged across the 8 active channels in each participant. *Z*-scores were computed using the mean and standard deviation of 20 surrounding frequency bins. A response was considered as face-selective if the Z-score at the frequency bin of face stimulation exceeded 3.09 (i.e., *p* < 0.001 one-tailed: signal > noise; Fig. S4).

### Reporting summary

Further information on research design is available in the Nature Portfolio Reporting Summary linked to this article.

### Supplementary information

Supplementary Information

Reporting Summary

### Source data

Source Data

## Data Availability

The data used in this study is available at: 10.25392/leicester.data.23459153.v1. Source data are provided with this paper.

## Code availability

Codes for data processing (spike sorting) are available at: https://github.com/csn-le/wave_clus.
