# Supplementary material for: Single neuron responses underlying face recognition in the human midfusiform face-selective cortex
Source: Nat Commun. 2023 Sep 13;14:5661. doi: 10.1038/s41467-023-41323-5 (PMC10499913; doi:10.1038/s41467-023-41323-5)
Supplement: Supplementary file 1 — Supplementary Information [file 41467_2023_41323_MOESM1_ESM.pdf]

Supplementary Information

Figure S1

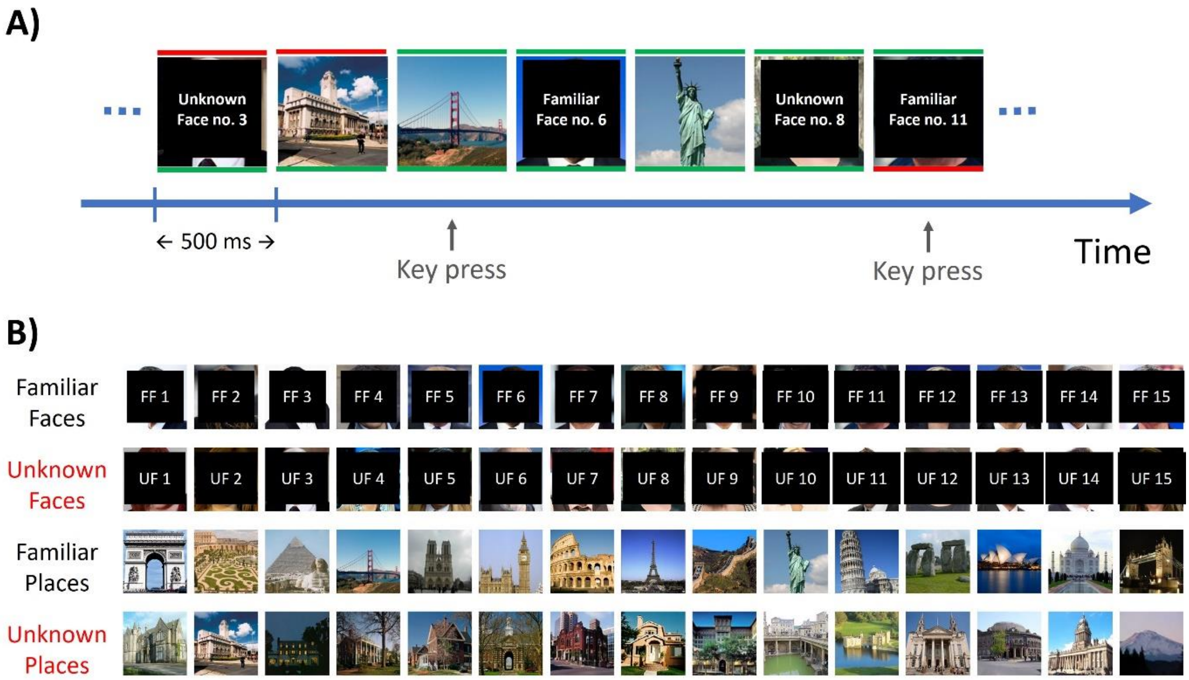

Figure S1: Paradigm design

A) Sequence of picture presentations. Pictures were shown for 500 ms each, in pseudorandom order and without blanks in between. To ensure they paid attention to the picture presentations, subjects were asked to press a key whenever one of the bars on top or at the bottom of the picture changed colour. B) Picture used in the experiment, corresponding to 4 stimulus categories: 15 familiar faces, 15 unknown faces, 15 familiar places and 15 unknown places. Face pictures are covered due to copyright issues.

Figure S2:

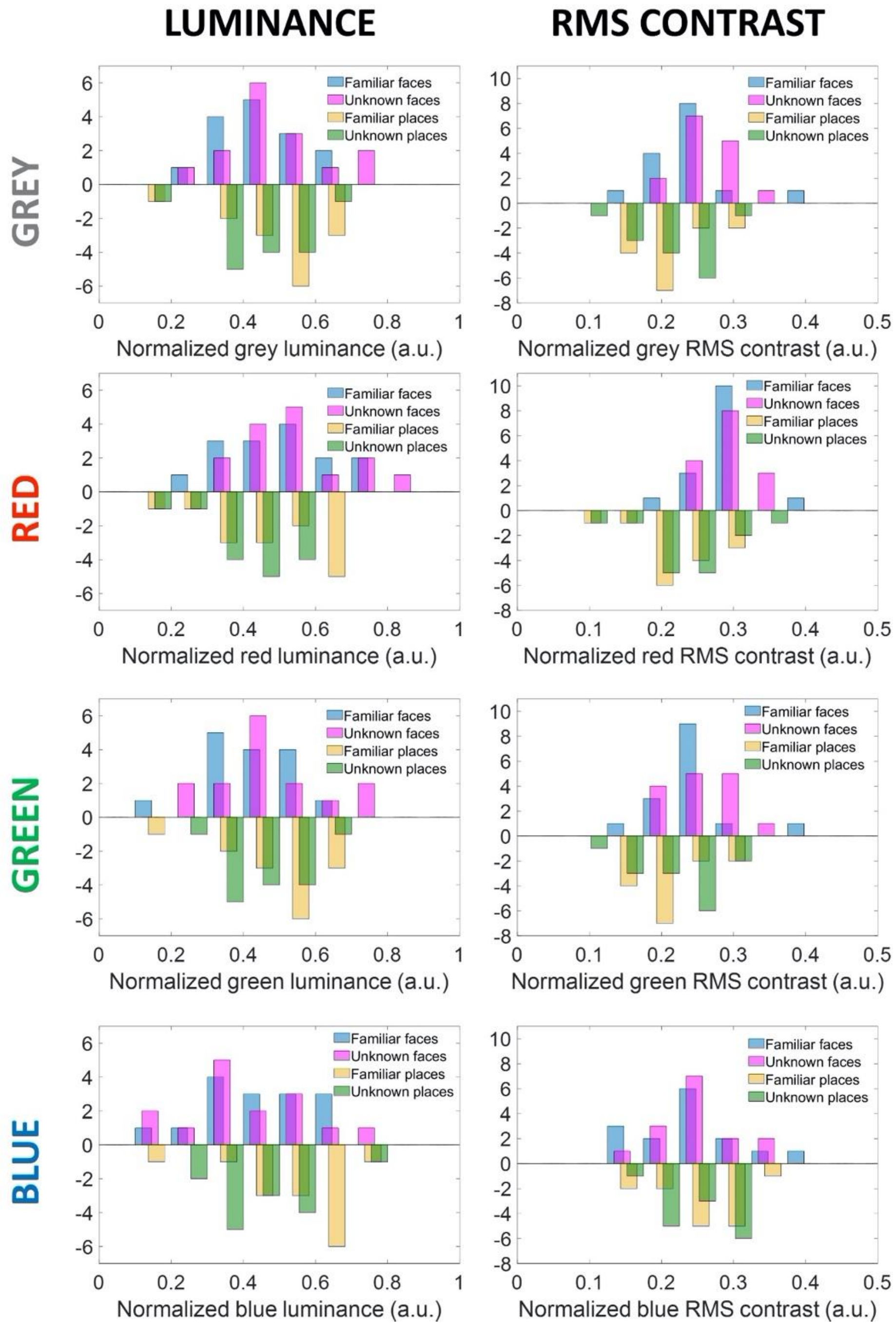

Figure S2: Low level visual features for the 4 stimulus categories.
The panels show the mean luminance and Root Mean Square contrast in greyscale and in the red, green and blue colours (RGB) for the 4 categories of stimuli. There were no significant differences for the luminance distributions and for the blue RMS contrast (ANOVA). However, the place pictures had significantly lower values for the RMS contrasts in greyscale, red and green, mainly due to the more homogeneous background of these images. To ensure that the neurons' responses could not be trivially attributed to the contrast of the images, we calculated the correlations between the neurons' responses and the contrast values both for the face and the place images, and verified that these were not significant—i.e. in less than 3 of the 43 responsive neurons a correlation was significant for the different contrast measures, which is within the range of what it is expected by chance (binomial test). (Left: bin size=0.1; Right: bin size=0.05)
Source data are provided as a Source Data file.

Figure S3:

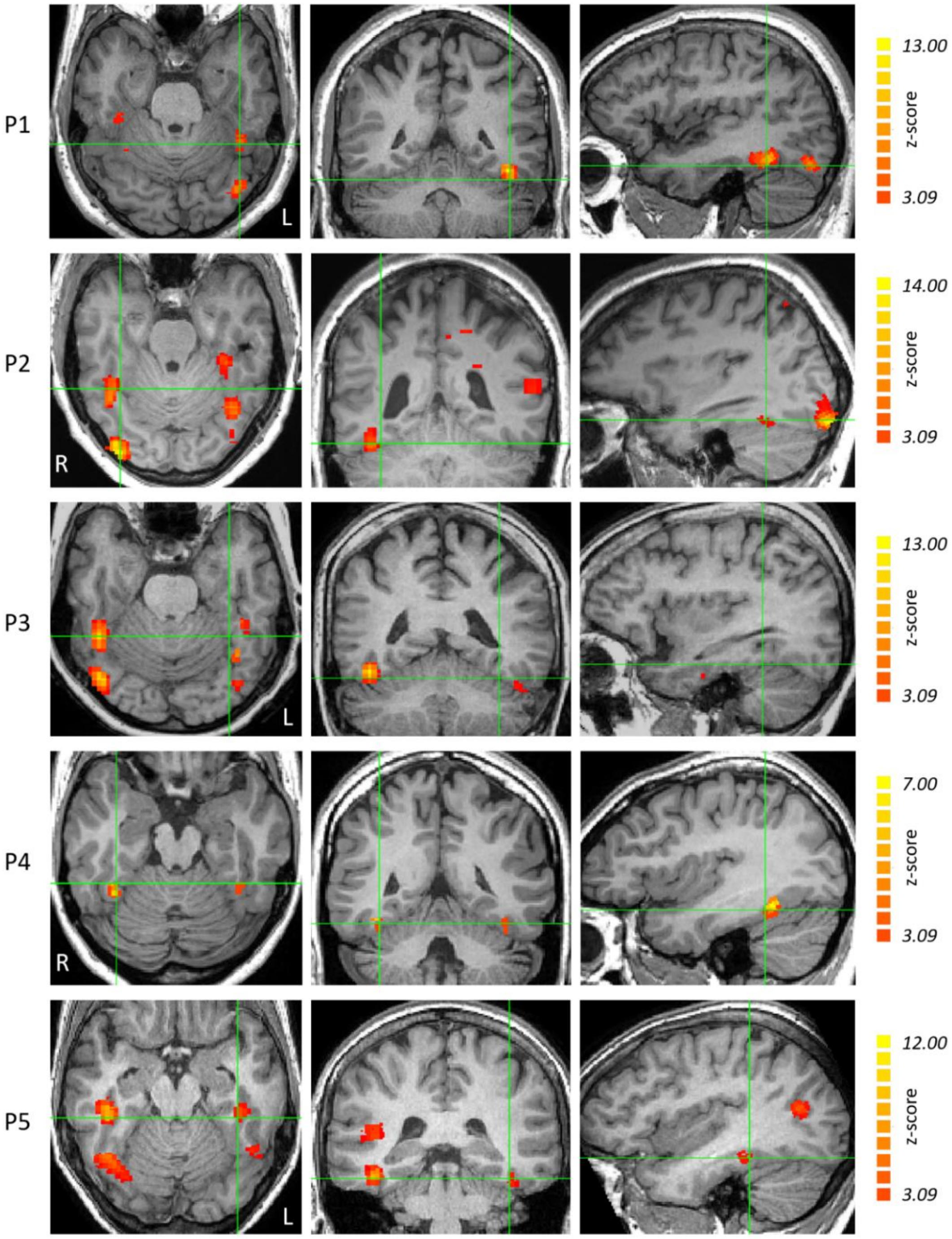

Figure S3: Location of fMRI face-selective responses together with the estimated position of the microwires (marked with a green cross, at about 3 mm from tip of the intracranial electrodes; see Fig. 1) for all patients. The colorbars on the right denote the normalized (z-score) activation compared to baseline. Note that in P2 and P3 the electrodes were slightly more medial and anterior, respectively, compared to the peak of the face-selective activations. However, in both cases the electrode ended approximately in the midfusiform gyrus, where significant fMRI face-selective activations were found (see Figure S4). Moreover, we obtained both face and place responses in all patients (Fig. S5), and the proportion of both face and place responses in patient 3 was statistically the same as for the other 4 patients (Fisher exact test, see Methods).

Source data are provided as a Source Data file.

Figure S4

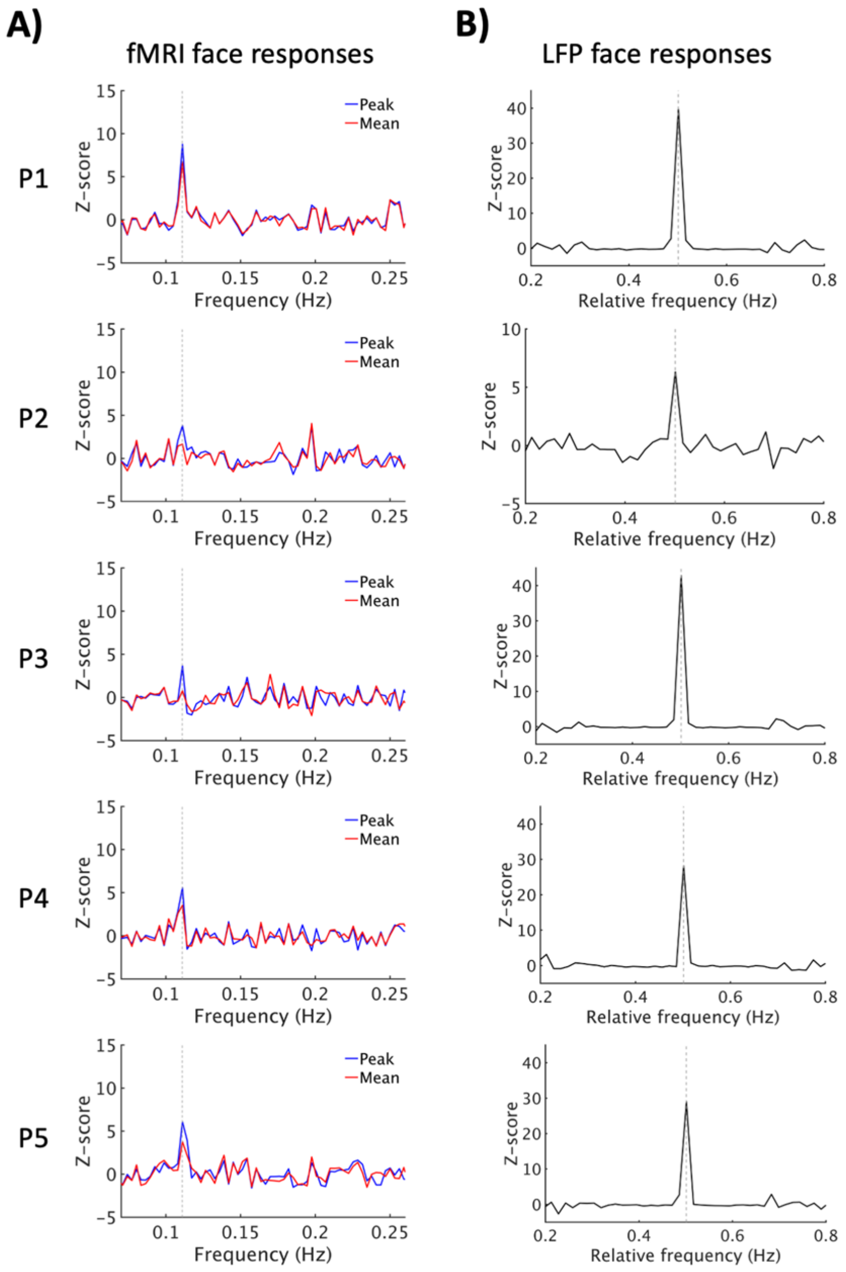

Figure S4: Face-selective responses at the electrodes' locations based on two independent "face-localizer" experiments. A) Normalized (z-score) face-selective fMRI response spectrum at the approximate location of the microwires (i.e. a 2mm ROI at 3mm from the tip of the intracranial electrode). Blue lines indicate the peak response spectrum within the ROI, and red lines indicate the mean response spectrum within the ROI. All patients showed face responses at 0.11 Hz (see Methods). B) Grand-averaged response SNR at the peak of the face-selective LFP responses (see Methods).

Source data are provided as a Source Data file.

Figure S5

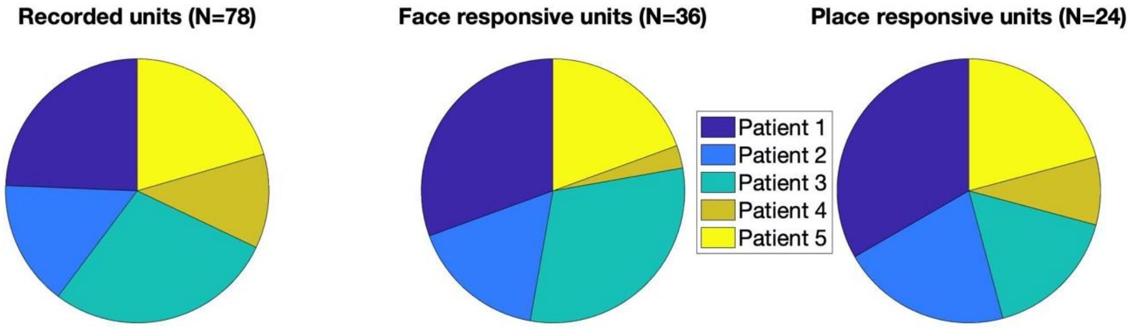

<u>Figure S5:</u> Distribution of recorded neurons (left), face responsive neurons (middle) and place responsive neurons (right) for all the patients.
Source data are provided as a Source Data file.

Figure S6

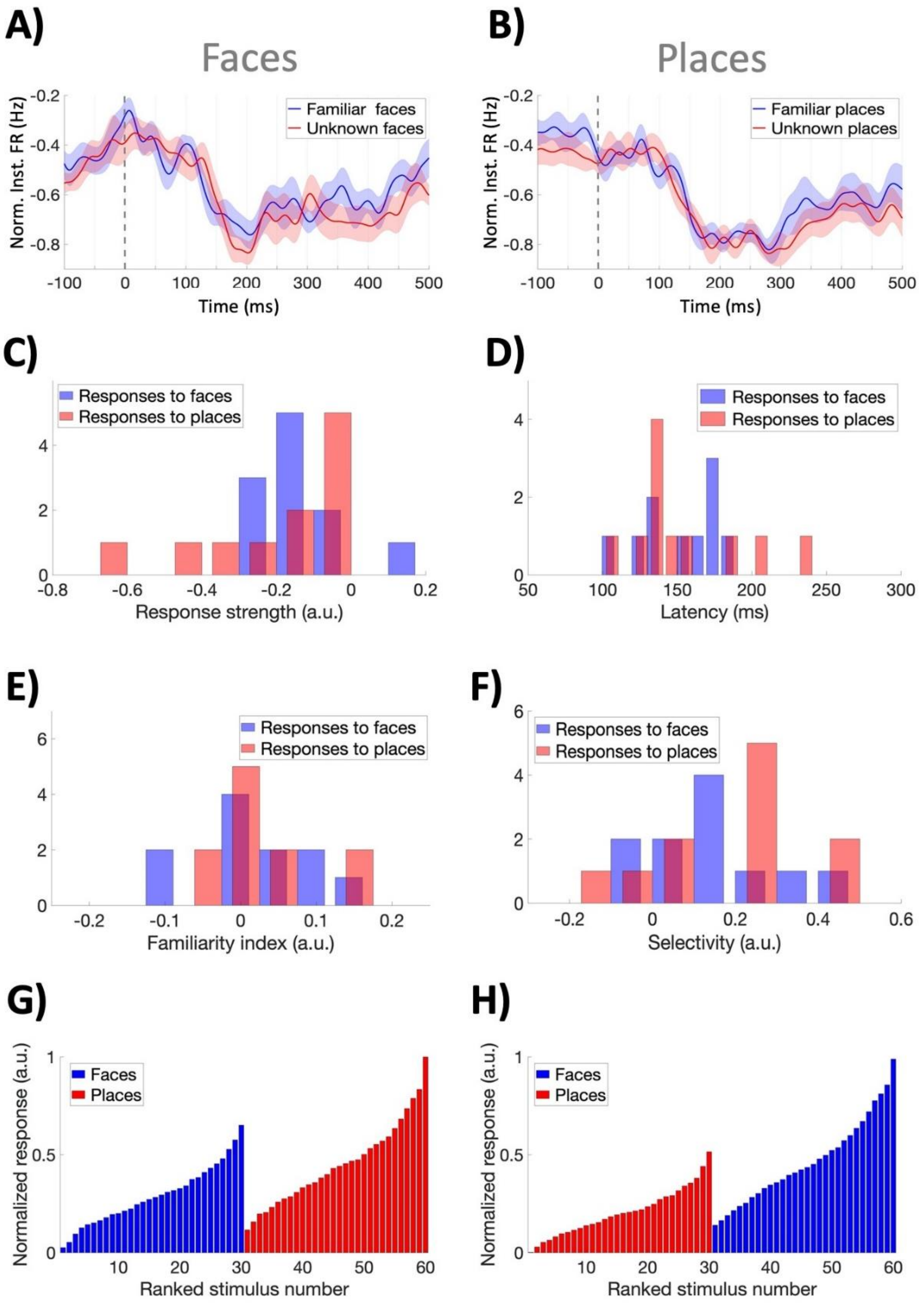

Figure S6: Characterization of suppressed responses.
A-H) Grand average responses and distributions of response strength, latency, familiarity index and selectivity for the suppressed responses, together with the ranked normalized suppressed responses. As for the enhanced responses, there were no significant differences between the face and place response distributions (C-F). Conventions are the same as in Figure 3. The shaded areas represent SEM. (C: bin size=0.1; D: bin size=10; E: bin size=0.05; F: bin size=0.1.)
Source data are provided as a Source Data file.

Figure S7

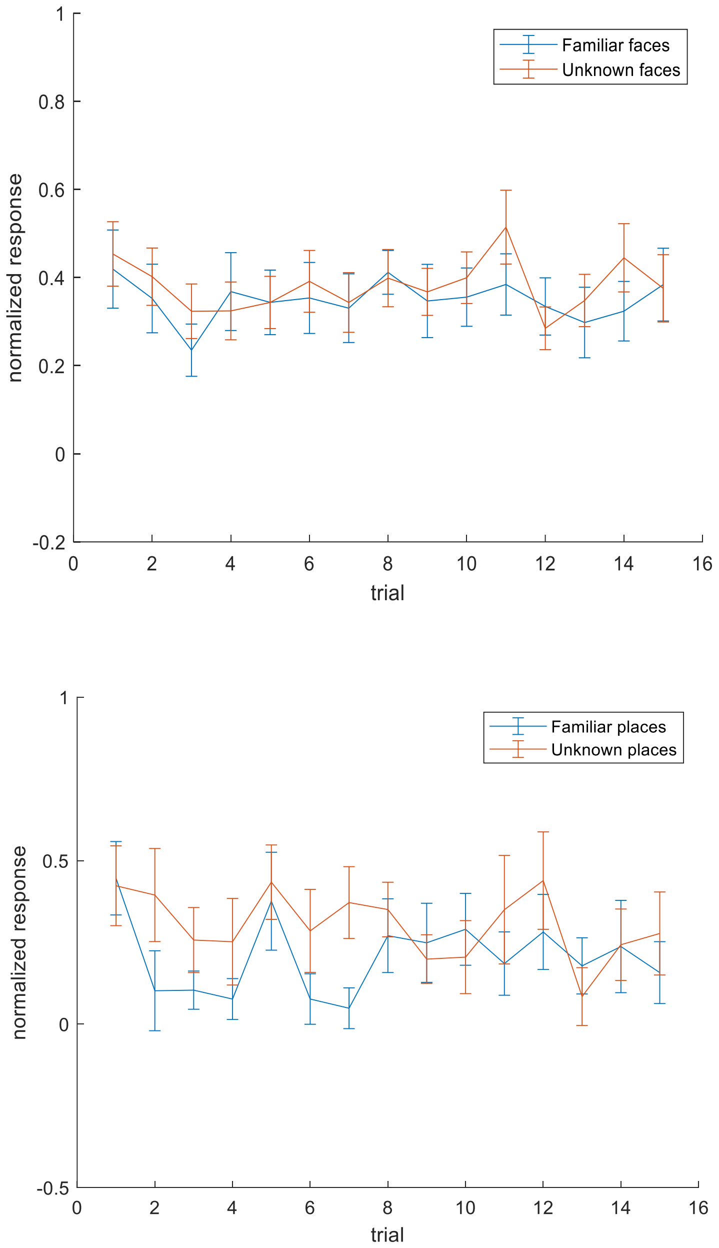

Figure S7: Study of potential adaptation or facilitation effects.
Normalized average responses as a function of trial number (the normalization was done subtracting the mean baseline activity and dividing by its s.d.), for the face (top; N=25) and place responses (bottom; N=13). Error bars denote SEM. There is no clear trend showing increases or decreases of the responses with trial number, signalling adaptation or facilitation effects.
Source data are provided as a Source Data file.

Figure S8

Faces vs. Places

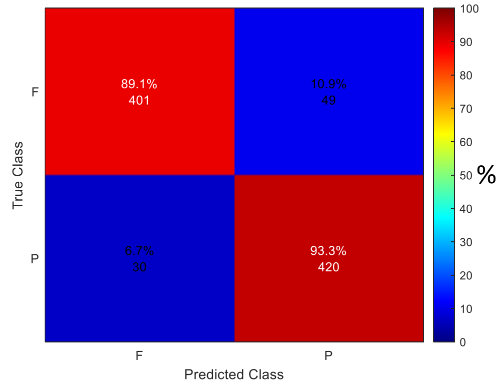

Familiar vs. Unknown Faces

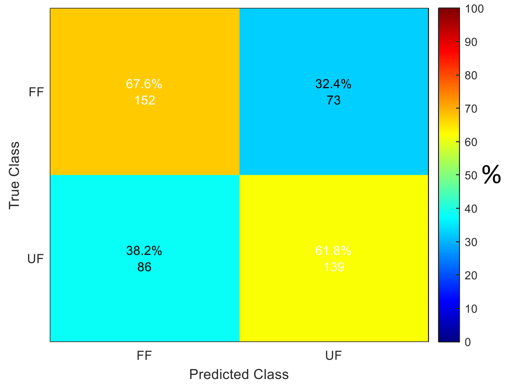

Familiar vs. Unknown Places

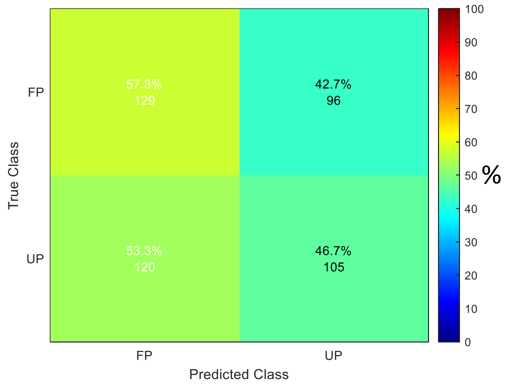

Figure S8: Decoding performances using a K-fold cross-validation.
Average decoding performances discriminating faces vs. places (top; permutation test, $p<10^{-91}$), familiar vs. unknown faces (middle; permutation test, $p<10^{-5}$), and familiar vs. unknown places (bottom; permutation test, p=0.25), using K-fold cross-validation, in which the trials of the particular face/place being tested were not included in the training set.
Source data are provided as a Source Data file.

Figure S9

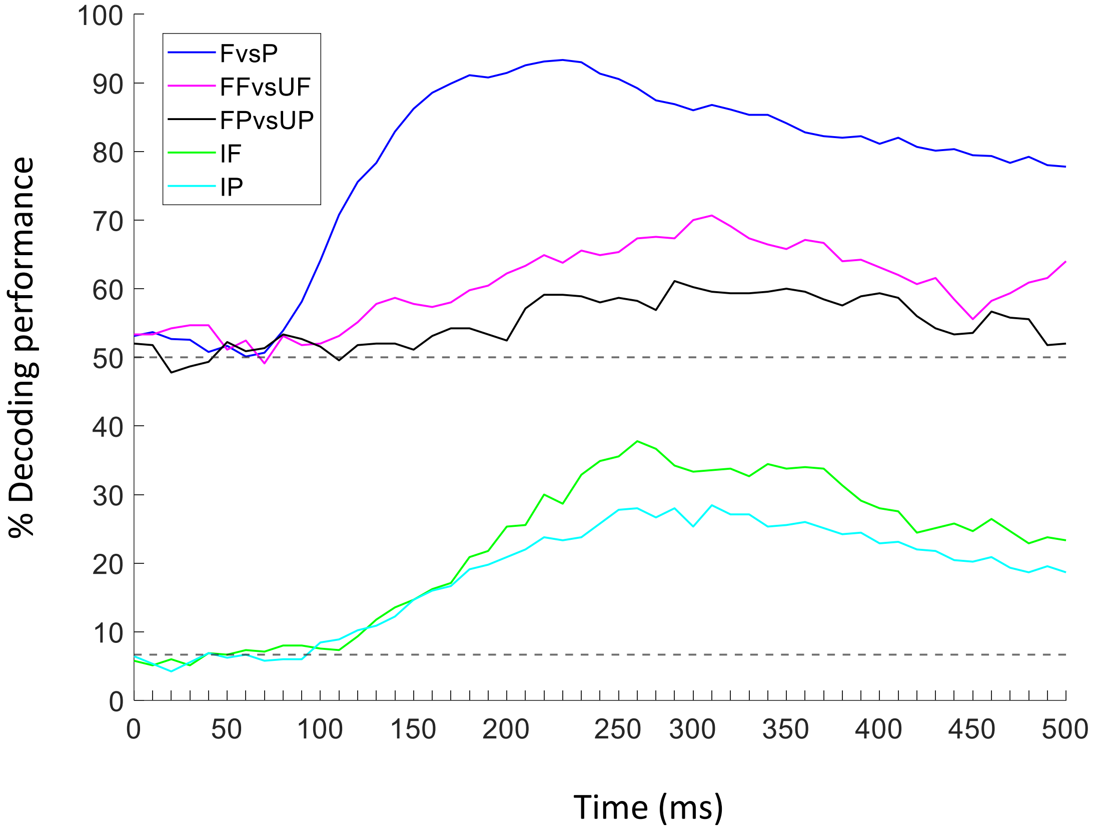

Figure S9: Time-resolved decoding performance.
Decoding performance obtained using a sliding window of 150 ms with 10 ms steps. Conventions are the same as in Figure 4 (F vs P: decoding of faces versus places; FF vs UF: decoding of famous versus unknown faces; FP vs UP: decoding of famous vs. unknown places; IF: decoding of individual face pictures and IP: decoding of individual place pictures). Results are similar to those obtained with the cumulative decoder (Fig 4e), showing an earlier (and stronger) identification of faces and places, followed by the identification of whether the faces (and to a lower degree places) are familiar or not.
Source data are provided as a Source Data file.

Figure S10

Faces vs. places

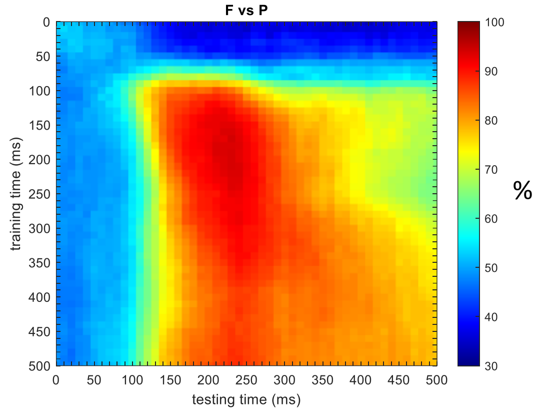

Famous vs. unknown faces (left) & famous vs. unknown places (right)

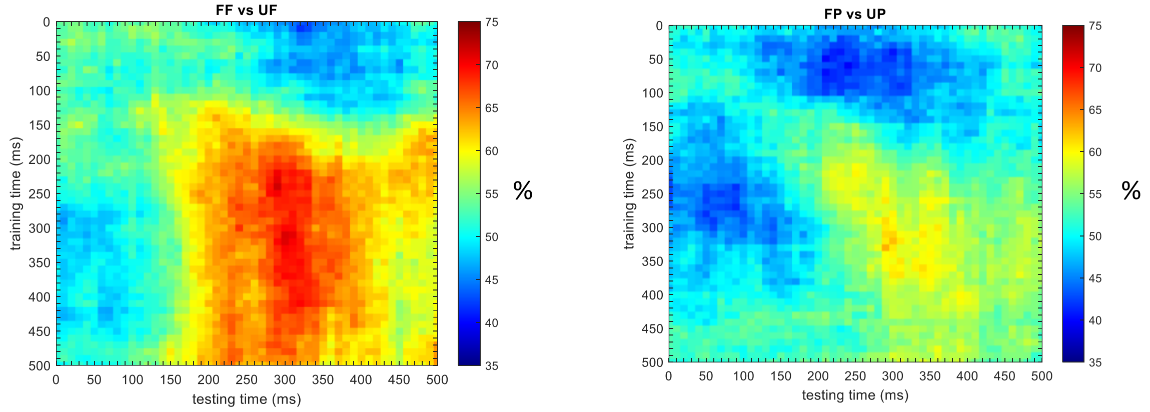

Individual face (left) and place (right) pictures

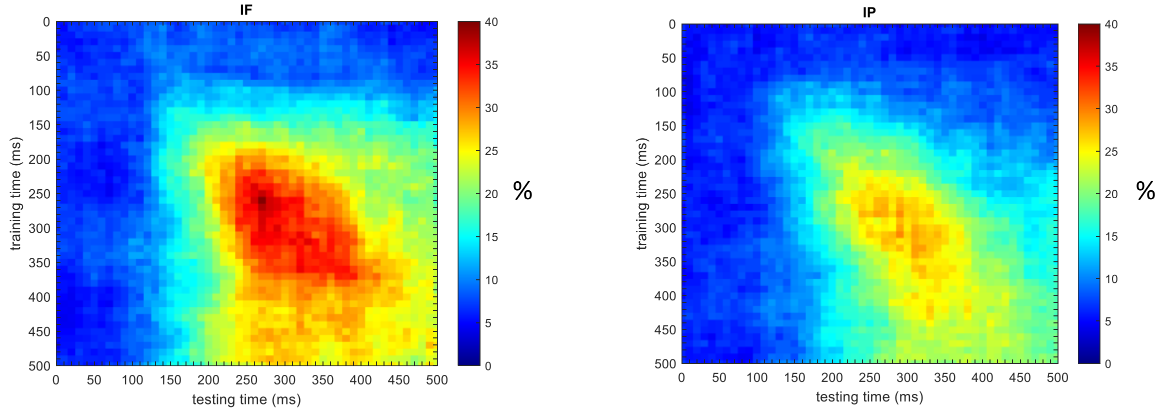

Figure S10: Cross-time decoding performance.
Cross-time decoding performance obtained using different time windows (with 150 ms width) for the training and testing sets. Results are qualitatively similar to those of Figure 4, showing a stronger, earlier and more sustained decoding of faces vs. places compared to a more transient and less strong decoding of familiar vs. unknown faces, a more transient decoding of the individual face pictures, and clearly lower decoding performances for discriminating between familiar vs. unknown places and the individual place pictures.

Source data are provided as a Source Data file.

Figure S11

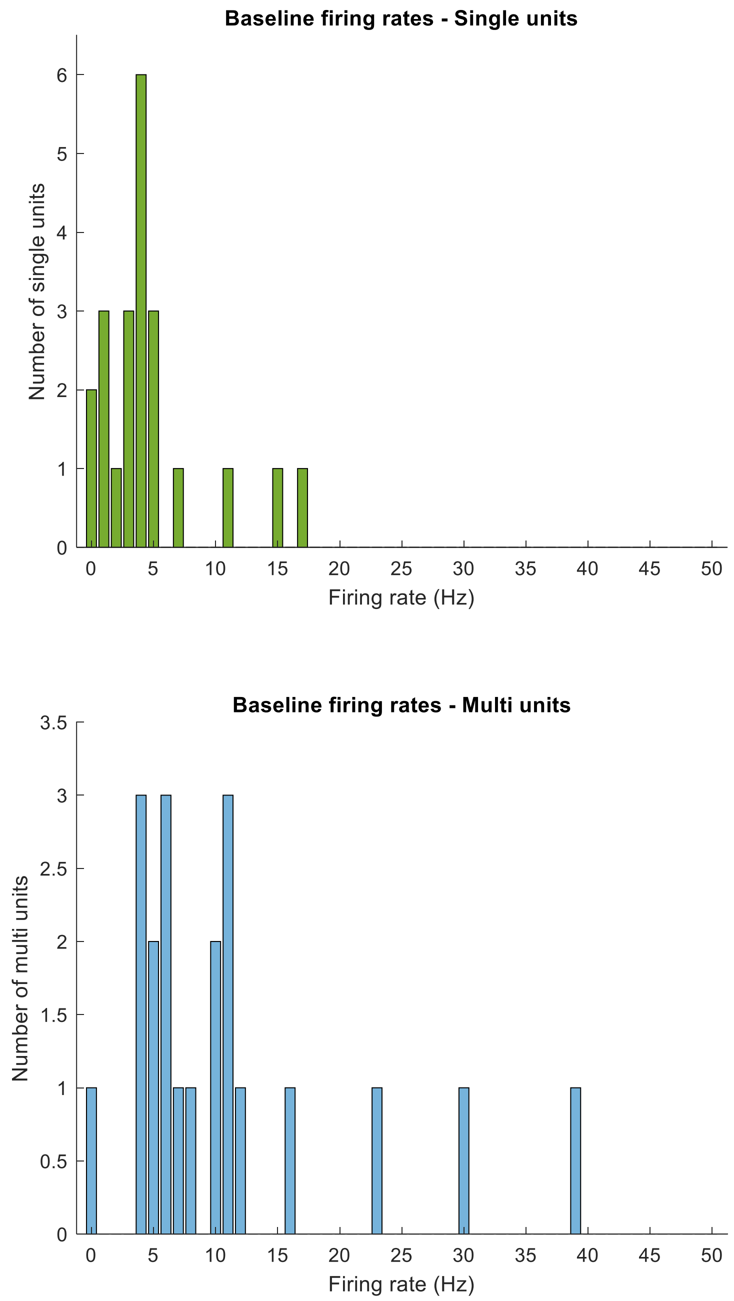

<u>Figure S11:</u> Distribution of baseline firing rates.
Baseline firing rates for the responsive single-units (top) and multi-units (bottom), calculated in a window from -100 to +100 ms from stimulus onset (n.b. the earliest responses were after 100 ms). The mean of the distributions was 4.8 Hz for the single-units and 10.9 Hz for the multi-units. Bin size is equal to 1.
Source data are provided as a Source Data file.

Table S1: Responsive units

	No response to Places	Excitatory resp. to Places	Inhibitory resp. to Places	
No response to Faces	35	5	2	
Excitatory resp. to Faces	14	3	8	25
Inhibitory resp. to Faces	5	5	1	11
		13	11	

Table S1: Number of non-responsive units (in grey) and of responsive units with excitatory responses to faces and places (total numbers in blue) and with inhibitory responses to faces and places (total numbers in red).
