# Supplementary material for: Single neuron responses underlying face recognition in the human midfusiform face-selective cortex
Source: Nat Commun. 2023 Sep 13;14:5661. doi: 10.1038/s41467-023-41323-5 (PMC10499913; doi:10.1038/s41467-023-41323-5)
Supplement: Supplementary file 2 — Reporting Summary [file 41467_2023_41323_MOESM2_ESM.pdf]

nature portfolio

Corresponding author(s): Rodrigo Quian Quiroga

Last updated by author(s): 19.6.23

Reporting Summary

Nature Portfolio wishes to improve the reproducibility of the work that we publish. This form provides structure for consistency and transparency in reporting. For further information on Nature Portfolio policies, see our Editorial Policies and the Editorial Policy Checklist.

Statistics

For all statistical analyses, confirm that the following items are present in the figure legend, table legend, main text, or Methods section.

n/a	Confirmed	
[ ]	[x]	The exact sample size (*n*) for each experimental group/condition, given as a discrete number and unit of measurement
[ ]	[x]	A statement on whether measurements were taken from distinct samples or whether the same sample was measured repeatedly
[ ]	[x]	The statistical test(s) used AND whether they are one- or two-sided<br>*Only common tests should be described solely by name; describe more complex techniques in the Methods section.*
[x]	[ ]	A description of all covariates tested
[ ]	[x]	A description of any assumptions or corrections, such as tests of normality and adjustment for multiple comparisons
[ ]	[x]	A full description of the statistical parameters including central tendency (e.g. means) or other basic estimates (e.g. regression coefficient) AND variation (e.g. standard deviation) or associated estimates of uncertainty (e.g. confidence intervals)
[ ]	[x]	For null hypothesis testing, the test statistic (e.g. *F*, *t*, *r*) with confidence intervals, effect sizes, degrees of freedom and *P* value noted<br>*Give P values as exact values whenever suitable.*
[ ]	[x]	For Bayesian analysis, information on the choice of priors and Markov chain Monte Carlo settings
[x]	[ ]	For hierarchical and complex designs, identification of the appropriate level for tests and full reporting of outcomes
[x]	[ ]	Estimates of effect sizes (e.g. Cohen's *d*, Pearson's *r*), indicating how they were calculated

Our web collection on statistics for biologists contains articles on many of the points above.

Software and code

Policy information about availability of computer code

Data collection	Data collection was done using the software provided by the data acquisition system (Blackrock; NeuroPort Central Suite version 6.5.4)
Data analysis	Spike sorting was used with Wave_Clus, an open source algorithm and all analyses were done in Matlab (R2018a).

For manuscripts utilizing custom algorithms or software that are central to the research but not yet described in published literature, software must be made available to editors and reviewers. We strongly encourage code deposition in a community repository (e.g. GitHub). See the Nature Portfolio guidelines for submitting code & software for further information.

Data

Policy information about availability of data

All manuscripts must include a data availability statement. This statement should provide the following information, where applicable:

- Accession codes, unique identifiers, or web links for publicly available datasets
- A description of any restrictions on data availability
- For clinical datasets or third party data, please ensure that the statement adheres to our policy

Data availability statement (with link to the repository where the data was placed) included in the paper.

Human research participants

Policy information about studies involving human research participants and Sex and Gender in Research.

Reporting on sex and gender	Number of male/female participants provided.
Population characteristics	Epilepsy patients that had to be implanted with intracranial electrodes. 5 patients (1 male, 4 females; all right-handed; 24-42 years old.
Recruitment	Patients were recruited based on clinical criteria - epilepsy patients candidate to epilepsy surgery. Inclusion to this project was based on the need, based on clinical criteria, to implant electrodes in the midfusiform gyrus. All patients implanted with verified implantation of the electrodes in the midfusiform gyrus were included in the study (no exclusion or selection biases were applied).
Ethics oversight	local ethical committee at the University Hospital of Nancy, France (CPP Est III, N°16.02.01)

Note that full information on the approval of the study protocol must also be provided in the manuscript.

Field-specific reporting

Please select the one below that is the best fit for your research. If you are not sure, read the appropriate sections before making your selection.

☒ Life sciences ☐ Behavioural & social sciences ☐ Ecological, evolutionary & environmental sciences

For a reference copy of the document with all sections, see nature.com/documents/nr-reporting-summary-flat.pdf

Life sciences study design

All studies must disclose on these points even when the disclosure is negative.

Sample size	Sample size was considered sufficient after evaluating the statistical results (significant effects with p<0.05 using the statistics described in the manuscript - pairwise T-test comparing baseline and response time windows). No statistical method was used to predetermine sample size.
Data exclusions	No exclusions were done.
Replication	All details are provided so that the data can be replicated. The data of the manuscript comes from 7 experiments performed in 5 subjects (2 experiments in 2 subjects and 1 experiment in the other 3 subjects). All experiments were sucessful at providing usable data and were included in the study.
Randomization	N/A. All patients were equally considered (i.e. there were not a 'control' vs. 'treament' comparisons), so there was no need of randomization.
Blinding	N/A. The analyses were done with codes that run automatically on the data, so there was no subjective bias issues and no need for double blinding.

Reporting for specific materials, systems and methods

We require information from authors about some types of materials, experimental systems and methods used in many studies. Here, indicate whether each material, system or method listed is relevant to your study. If you are not sure if a list item applies to your research, read the appropriate section before selecting a response.

Materials & experimental systems

n/a	Involved in the study	
☒	☐	Antibodies
☒	☐	Eukaryotic cell lines
☒	☐	Palaeontology and archaeology
☒	☐	Animals and other organisms
☒	☐	Clinical data
☒	☐	Dual use research of concern

Methods

n/a	Involved in the study	
☒	☐	ChIP-seq
☒	☐	Flow cytometry
☐	☒	MRI-based neuroimaging

Magnetic resonance imaging

Experimental design

Design type	Face localizer experiment
Design specifications	Presentation of face vs. object pictures to localize face selective areas
Behavioral performance measures	Passive viewing of the pictures

Acquisition

Imaging type(s)	MRI, fMRI
Field strength	3T
Sequence & imaging parameters	T1-weighted magnetization-prepared gradient-echo image (MP-RAGE) sequence (192 sagittal slices, voxel size = 1 mm isotropic; flip angle (FA) = 9 º, field of view (FOV) = 256 × 256 mm2, matrix size = 256 × 256); T2*-weighted simultaneous multi-slice echo planar imaging (SMS EPI) sequence (TR = 1500 ms, TE = 34 ms, FA = 72º, FOV= 240×240 mm2, voxel size = 2.5 mm isotropic, matrix size = 96×96, interleaved), which acquired 44 oblique-axial slices
Area of acquisition	whole brain

Diffusion MRI ☐ Used ☒ Not used

Preprocessing

Preprocessing software	MCFLIRT; FSL
Normalization	Data not normalized (analyses in individual brain space)
Normalization template	Data not normalized
Noise and artifact removal	motion-corrected with reference to the average image of the first functional run using a 6-degree rigid body translation and rotation using a motion correction software (MCFLIRT; www.fmrib.ox.ac.uk/fsl), and spatially smoothed with a Gaussian kernel of 3 mm (FWHM; i.e. full width at half maximum). Linear trends from the preprocessed time series of each voxel were removed.
Volume censoring	The functional runs were motion-corrected with the mean image of the first experimentl run with 6 degrees of rigid body translation and rotation via an intra-modal volume linear registration with the FMRIB Software Library (FSL, version 5.0.8, Smith et al. 2004).

Statistical modeling & inference

Model type and settings	Univariate. Model-free analysis: Fourier Transform (Gao et al., 2018)
Effect(s) tested	Only face-selective responses measured.

Specify type of analysis: ☐ Whole brain ☒ ROI-based ☐ Both

Anatomical location(s): Functional ROIs

Statistic type for inference (See Eklund et al. 2016)	Cluster-wise. Response was considered as face-selective if the Z-score at the frequency bin of face stimulation exceeds 3.09 (i.e., p < .001 one-tailed: signal>noise).
Correction	We do not test the whole brain or a large region of interest. Instead, we have a highly constrained hypothesis to test a small volume around the estimated position of the microelectrodes (with a minimum of 10 2.5x2.5x2.5 mm3 voxels), so there is no need for multiple comparison correction. Note that a stringent criterion, with p<0.001 (all 5 individual Z-scores for the peak > 3.09), was used.

Models & analysis

n/a	Involved in the study
☒	☐ Functional and/or effective connectivity
☒	☐ Graph analysis
☒	☐ Multivariate modeling or predictive analysis
